# Occurrence of *mcr*-mediated colistin resistance in *Salmonella* clinical isolates in Thailand

**DOI:** 10.1038/s41598-021-93529-6

**Published:** 2021-07-08

**Authors:** Sirirat Luk-in, Tanittha Chatsuwan, Naris Kueakulpattana, Ubolrat Rirerm, Dhammika Leshan Wannigama, Rongpong Plongla, Ratana Lawung, Chaiwat Pulsrikarn, Siriporn Chantaroj, Pattharaporn Chaichana, Nattida Saksaengsopa, Teerarat Shanthachol, Malee Techapornroong, Sunee Chayangsu, Wanla Kulwichit

**Affiliations:** 1 Department of Microbiology, Faculty of Medicine, Chulalongkorn University, Bangkok, Thailand; 2 Department of Clinical Microbiology and Applied Technology, Faculty of Medical Technology, Mahidol University, Nakhon Pathom, Thailand; 3 Antimicrobial Resistance and Stewardship Research Unit, Faculty of Medicine, Chulalongkorn University, Bangkok, Thailand; 4 Department of Medicine, Faculty of Medicine, Chulalongkorn University and King Chulalongkorn Memorial Hospital, Thai Red Cross Society, Bangkok, Thailand; 5 School of Medicine, Faculty of Health and Medical Sciences, The University of Western Australia, Nedlands, WA Australia; 6 Salmonella and Shigella Center, National Institute of Health, Department of Medical Sciences, Nonthaburi, Thailand; 7 Department of Medical Sciences, Medical Science Center Khon Kaen, Nonthaburi, Thailand; 8 Queen Savang Vadhana Memorial Hospital, Thai Red Cross Society, Chonburi, Thailand; 9 Department of Medicine, Prapokklao Hospital, Chanthaburi, Thailand; 10 Department of Internal Medicine, Surin Hospital, Surin, Thailand

**Keywords:** Antimicrobials, Bacteria, Bacterial genes

## Abstract

Nontyphoidal *Salmonella*, an important zoonotic pathogen and a major cause of foodborne illnesses, could be a potential reservoir of plasmids harbouring mobile colistin resistance gene (*mcr*). This study reported, for the first time, a high rate of *mcr*-carrying *Salmonella* clinical isolates (3.3%, 24/724) in Thailand, associated with *mcr-3* gene (3.0%, 22/724) in *S.* 4,[5],12:i:-(15.4%, 4/26), *S*. Typhimurium (8.8%, 5/57), and *S*. Choleraesuis (5.6%, 13/231). Remarkably, the increasing trends of colistin and extended-spectrum cephalosporin resistances have displayed a high agreement over the years, with a dramatic rise in the *mcr*-carrying *Salmonella* from 1.1% (6/563) during 2005–2007 to 11.2% (18/161) during 2014–2018 when CTX-M-55 became abundant. Clonal and plasmid analysis revealed that the self-transferable IncA/C and a novel hybrid IncA/C-FIIs MDR plasmids were the major vehicles to disseminate both *mcr-3* and *bla*_CTX-M55_ genes among diverse *Salmonella* strains, from as early as 2007. To our knowledge the occurrence of *mcr-3* and the co-existence of it with *bla*_CTX-M-55_ in *S.* Choleraesuis are reported here for the first time, leading to clinical concern over the treatment of the invasive salmonellosis. This study provides evidence of the potential reservoirs and vectors in the dissemination of the *mcr* and highlights the co-selection by colistin and/or cephalosporins.

## Introduction

Antimicrobial resistance (AMR) is the greatest public health threat that can cross between human and other animal populations. This problem requires action at global, regional, and national levels to tackle it. Recently, the World Health Organisation published a catalogue of 12 families of antimicrobial-resistant bacteria that pose the greatest threat to human health^1^. These bacteria are resistant to a large number of antimicrobials, especially to carbapenems and extended-spectrum cephalosporins (ESCs) that are the best available antibiotics for treating multi-drug resistant (MDR) Gram-negative bacteria. This then leads to the use of colistin, a widely used antimicrobial in animal production for decades, as one of the last-resort therapeutic options^2^. Currently, colistin resistance has become of significant concern, due to the identification of plasmid-mediated colistin resistance, conferred by the mobile colistin resistance (*mcr*) gene.

The *mcr-1* gene was first identified in *Escherichia coli* mainly in swine isolates (> 20%) during 2011 to 2014^3^. Nowadays, ten *mcr*-like genes (*mcr-1* to *mcr-10*) have been reported among human and animal bacterial isolates^3–12^. Recently, a high frequency of *mcr* genes was reported in nontyphoidal *Salmonella*, an important zoonotic pathogen and a major cause of foodborne illnesses in many countries, including China^13–15^, Taiwan^16^, France^17^, Germany^17^, and Portugal^18,19^. Interestingly, at least six types of *mcr* genes (*mcr-1* to *-5* and *mcr-9*)^4,6,7,16,20,21^ have been identified in many serotypes of *S. enterica*, including *S.* Typhimurium^16,22^, *S.* Rissen^20^, *S.* 1,4,[5,12:i:-^20^, *S.* Anatum^16^, *S.* Albany^16^, *S.* Newport^16^, *S.* Derby^22^, *S.* Indiana^22^, and *S.* London^22^. In addition, at least three types of *mcr* genes (*mcr-4*, *-5*, and *-9*) had their first discovery in a *S. enterica* strain^6,7,11^. However, in contrast to the extensive studies focused on *E. coli*, there is only limited data available for *mcr*-harbouring *Salmonella* isolates and data on the plasmids carrying *mcr* genes responsible for the spread of colistin resistance.

Moreover, the *mcr* genes have been described in diverse genetic environments, on a wide variety of plasmid types, including the IncI2, IncHI2, and IncX4 plasmids^13–16,18,19^, especially in animal isolates. Notably, the co-occurrence of *mcr* genes and extended-spectrum beta-lactamases (ESBLs) have been reported since 2016 from *E. coli* calf isolates in France^23^ and continuously recognised among both *E. coli* and *Salmonella* isolates, leading to the assumed relationship between the ESBLs and *mcr* gene^21,23–26^. Considering that the combination of these genes could speed up the dissemination of MDR and extensively drug resistance (XDR) among Gram-negative bacteria, information regarding the co-occurrence and emergence of *mcr* and ESBL genes among the isolates are needed.

Here, we aimed to assess the dissemination mechanism of colistin resistance among *Salmonella* isolates, and the co-occurrence of *mcr* and ESBL genes in *Salmonella* clinical isolates in Thailand, the AMR mechanisms, clonality, and plasmid profiles of the isolates, as well as the transferability and characteristics of the resistance plasmids were investigated.

## Results

### Bacterial strains and antimicrobial susceptibility

A total of 724 nontyphoidal *Salmonella* isolates were recovered from the clinical samples collected from 32 out of 77 provinces in Thailand during the two periods of 2005–2007 (n = 563) and 2014–2018 (n = 161). More than 40 serotypes were identified from these isolates, most of which were *S.* Enteritidis (43.0%, 311/724), followed by *S.* Choleraesuis (31.9%, 231/724), *S.* Typhimurium (7.9%, 57/724), and *S.* 4,5,12:I:-(3.6%, 26/724). A total of 422 isolates (58.3%) were from bacteremic patients, most of which were *S.* Choleraesuis (46.2%, 195/422) and *S.* Enteritidis (43.4%, 183/422). Overall, a high rate of AMR was found among 724 nontyphoidal *Salmonella* isolates from patients in Thailand. Most isolates (75.3%, 545/724) were resistant to at least three classes of antimicrobial agents, whereas less than 5% of isolates (35/724) were susceptible to all antimicrobial agents tested. The most common resistance was to nalidixic acid (84.9%, 615/724), followed by ampicillin (77.4%, 560/724), tetracycline (50.6%, 366/724), chloramphenicol (32.0%, 232/724), gentamicin (26.9%, 195/724), trimethoprim-sulfamethoxazole (20.9%, 151/724), and ciprofloxacin (10.9%, 79/724). A high frequency of ESC-resistant *Salmonella* was observed in 22.9% of isolates (166/724), most of which were *S.* Choleraesuis (78.9%, 131/166). Of the 166 ESC-resistant isolates, 125 isolates (75.3%) were isolated from bacteremic patients. Resistance to ertapenem was not found in this study. Colistin susceptibility of nontyphoidal *Salmonella* clinical isolates in Thailand revealed a minimum inhibitory concentration (MIC) of colistin ranging from 0.25–32 µg/mL. Of note, a high rate of colistin resistance (18.8%, 136/724) with MIC range of 4–32 µg/mL was identified among the isolates.

**Figure 1:**
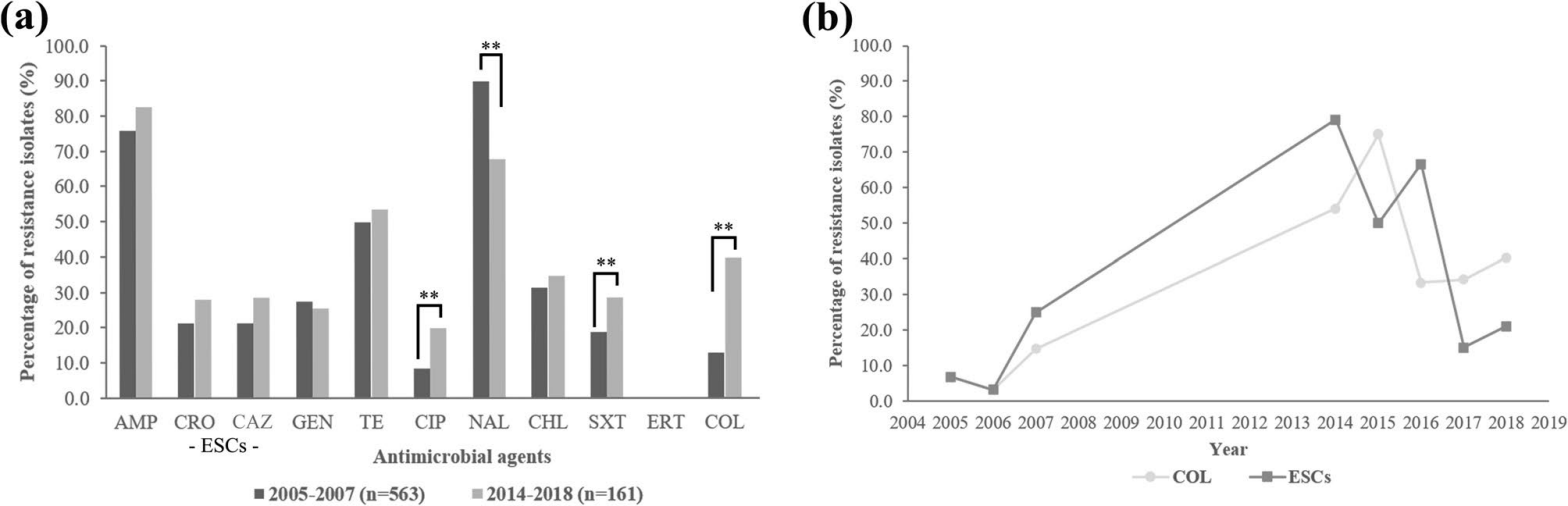
The AMR among 724 nontyphoidal *Salmonella* isolated from humans in Thailand during 2005–2007 and 2014–2018. (**a**) Comparison of AMR frequency in nontyphoidal *Salmonella* isolates between 2005–2007 and 2014–2018. (**b**) Frequency of ESC resistance and colistin resistance over time for nontyphoidal *Salmonella* isolated from patients from 2005–2016. *AMP* ampicillin, *CRO* ceftriaxone, *CAZ* ceftazidime, *GEN* gentamicin, *TE* tetracycline, *CIP* ciprofloxacin, *NAL* nalidixic acid, *CHL* chloramphenicol, *SXT* trimethoprim-sulfamethoxazole, *ERT* ertapenem, *COL* colistin, *ESCs* extended-spectrum cephalosporins.

The AMR frequency in *Salmonella* isolates was considerably higher in those isolated during 2014–2018 than in 2005–2007 for all tested antimicrobial agents except for nalidixic acid and gentamicin (Fig. 1a). Notably, during those periods, a significant increase in the frequency of colistin resistance from 12.8% (72/563) to 39.8% (64/161) of isolates (p < 0.0001) was found between these two time periods. Over 2005–2018, the frequency of colistin resistance increased as follows: 2005 (6.7%, 5/74), 2006 (3.1%, 1/32), 2007 (14.7%, 67/457), 2014 (54.2%, 13/24), 2015 (75.0%, 3/4), 2016 (33.3%, 1/3), 2017 (34.3%, 25/73), and 2018 (40.4%, 23/57). We found that the increasing trend of colistin resistance and ESC resistance frequencies displayed a high agreement with each other over those time periods (Fig. 1b). Remarkably, an increasing frequency of *mcr*-carrying isolates was found from 1.1% (6/563) in 2005–2007 to 11.2% (18/161) in 2014–2018 (Supplementary Fig. S1).

**Figure 2:**
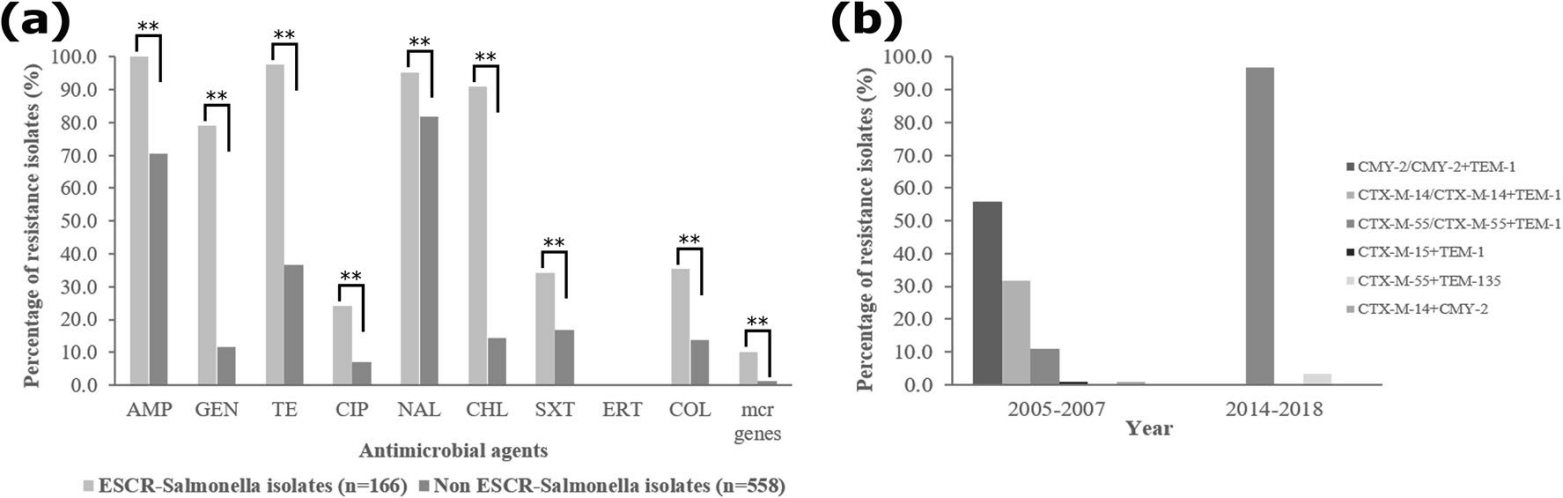
(**a**) Comparison of the AMR observed between ESC-resistant *Salmonella* and non ESC-resistant counterparts from the 724 nontyphoidal *Salmonella* isolated from patients in Thailand during 2005–2007 and 2012–2016. (**b**) Resistance mechanisms detected among ESC-resistant *Salmonella* isolates in 2005–2007 and 2014–2018. *AMP* ampicillin, *GEN* gentamicin, *TE* tetracycline, *CIP* ciprofloxacin, *NAL* nalidixic acid, *CHL* chloramphenicol, *SXT* trimethoprim-sulfamethoxazole, *ERT* ertapenem, *COL* colistin, *ESCs* extended-spectrum cephalosporins; *, *p* < 0.05; and **, *p* < 0.01.

Comparison of the AMR between ESC-resistant *Salmonella* and non-ESC-resistant *Salmonella* isolates from the whole strain collection revealed that ESC-resistant *Salmonella* isolates displayed significantly higher resistance frequencies in all the tested antimicrobial agents (p < 0.0001) than in the non-ESC-resistant *Salmonella* isolates (Fig. 2a). Notably, we found that ESC-resistant *Salmonella* isolates had significantly higher rates of colistin resistance (35.5% (59/166) vs. 13.8% (77/558), p < 0.0001) and the presence of *mcr* genes (10.2% (17/166) vs. 1.3% (7/558), p < 0.0001) than in the non-ESC-resistant *Salmonella* isolates (Fig. 2a). In addition, among the ESC-resistant *Salmonella* isolates, a significant increase in the frequency of *mcr*-harbouring isolates from 5% of the isolates (6/120) in 2005–2007 to 23.9% (11/46) in 2014–2017 (*p* < 0.0001) was detected. During 2005–2007, CMY-2 (55.8%, 67/120) was the most common mechanism of ESC resistance detected among the ESC-resistant isolates. The emergence of CTX-M-55 was detected in all 46 ESC-resistant *Salmonella* isolates during 2014–2018, one of which displayed both CTX-M-55 and TEM-135 (Fig. 2b).

Of the 24 *mcr*-harbouring *Salmonella* isolates, 22 isolates carried *mcr-3* variant genes (21 for *mcr-3.1* and one for *mcr-3.2*) with a colistin MIC range from 4–32 μg/mL, and two isolates carried the *mcr-1.9* gene with a colistin MIC of 8 μg/mL. Markedly, of the 166 ESC-resistant *Salmonella* isolates, 17 carried the *mcr-3* gene, and 15 and two isolates co-harboured the *mcr-3* and *bla*_CTX-M55_ or *bla*_CMY-2_ gene, respectively. The high prevalence of *mcr-3* genes was found in *S.* Typhimurium and *S.* 4,5,12:I:-, a monophasic variant of *S.* Typhimurium, with 8.8% (5/57) and 15.4% (4/26) of isolates, respectively. Moreover, the *mcr-3.1* gene was detected in 5.6% of *S.* Choleraesuis isolates (13/231), all of which were from bacteremic patients and most of which co-harboured both *bla*_CTX-M55_ and *qnrS1* genes.

### Clonal relatedness

**Figure 3:**
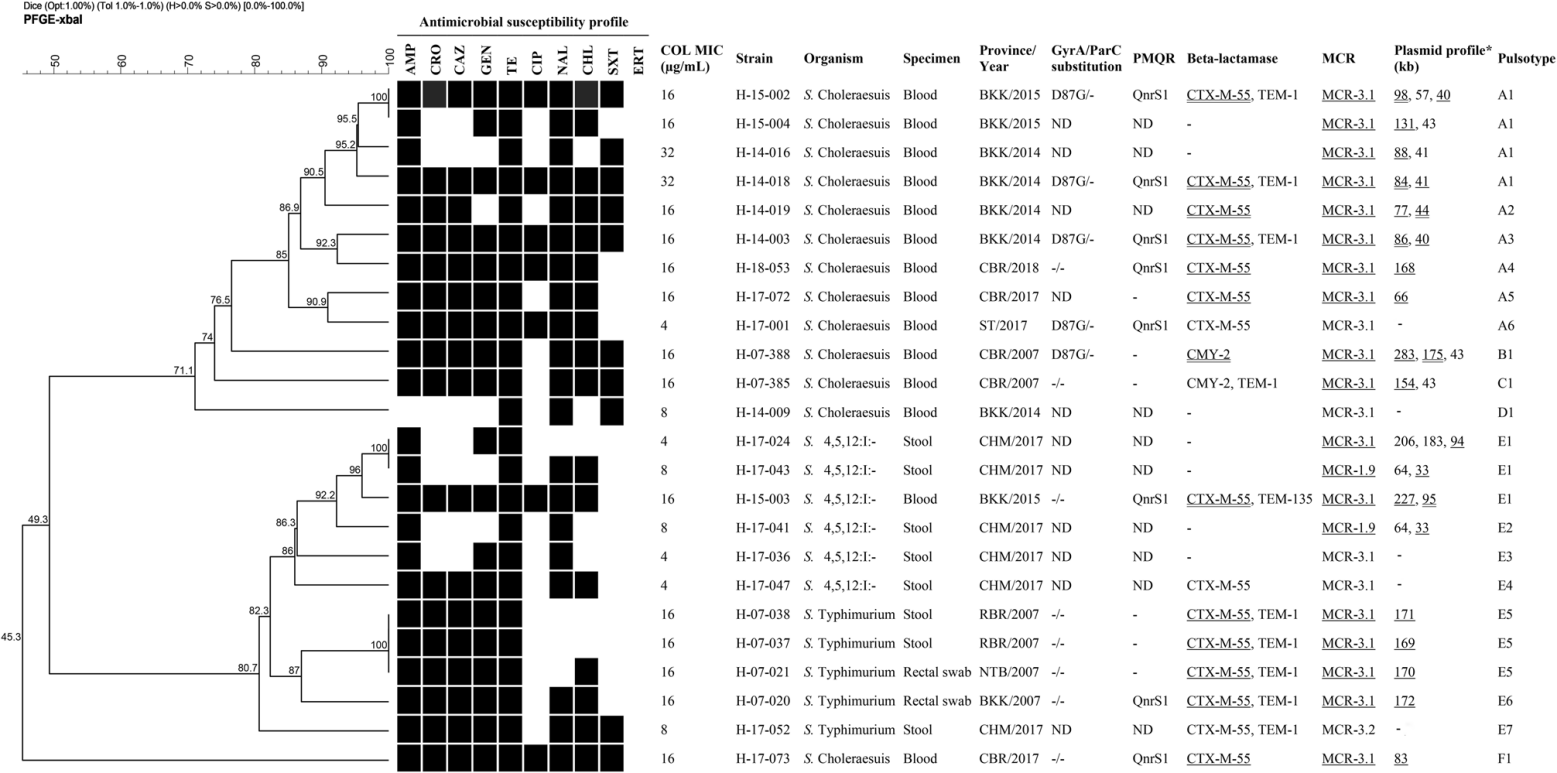
Dendrogram generated by PFGE-*Xba*I of 24 *mcr*-harbouring *Salmonella* isolates from humans in Thailand. Isolate summary information showing antimicrobial susceptibility profiles, colistin MIC, AMR mechanisms, plasmid profiles, and pulsotypes. Black squares represent isolates that were resistant to antimicrobial agents. Antimicrobial agents are abbreviated as follows: *AMP* ampicillin, *CRO* ceftriaxone, *CAZ* ceftazidime, *GEN* gentamicin, *TE* tetracycline, *CIP* ciprofloxacin, *NAL* nalidixic acid, *CHL* chloramphenicol, *SXT* trimethoprim-sulfamethoxazole, *ERT* ertapenem, *COL* colistin, *BKK* Bangkok, *CBR* Chonburi, *ST* Satun, *CHM* Chiang Mai, *RBR* Ratchaburi, *NTB* Nonthaburi, *PMQR* plasmid-mediated quinolone resistance, *ND* not determined; and -, not found. *Plasmid sizes and AMR mechanisms confirmed for the location on the plasmid by Southern blot and hybridisation are underlined by a single line or a double line.

All 24 *mcr*-harbouring *Salmonella* isolates (13 *S.* Choleraesuis, 5 *S.* Typhimurium, and 6 *S.* 4,5,12:I:-) were subtyped by PFGE, revealing that they formed six PFGE clusters (designated A to F) and 17 different pulsotypes, when using a cut-off of 80% and 95% genetic similarity, respectively (Fig. 3). The most common pulsotype (A1) contained four *mcr3.1*-carrying *S.* Choleraesuis isolates obtained from four different bacteremic patients in Bangkok during 2014–2015, two of which displayed a high-level colistin MIC (16 and 32 μg/mL, respectively) and were resistant to all antimicrobial agents tested except for ertapenem. These isolates had the D87G amino acid substitution in GyrA and carried the *bla*_CTX-M55_, *bla*_TEM-1_, and *qnrS1* genes. Pulsotype E1, a major pulsotype among the *S.* 4,5,12:I:- isolates, contained three *mcr*-harbouring *S.* 4,5,12:I:- isolates (two of *mcr-3.1* and one of *mcr-1.9*) from two different provinces (Bangkok and Chiang Mai) in 2015 and 2017. One *mcr3.1*-carrying *S.* 4,5,12:I:- blood isolate was resistant to all antimicrobial agents tested except for ertapenem. In addition, this isolate also carried some other plasmid-borne AMR determinants, including *bla*_CTX-M55_, *bla*_TEM-135_, and *qnrS1* genes. The most common pulsotype among the *mcr*-harbouring *S.* Typhimurium isolates, pulsotype E5, contained three indistinguishable *mcr-3.1*-carrying *S.* Typhimurium isolates obtained from stool and rectal swab from two different provinces (Ratchaburi and Nonthaburi) in Thailand in 2007. These isolates displayed an ESC-resistance phenotype that was mediated by the *bla*_CTX-M55_ and *bla*_TEM-1_ genes.

### Plasmid characterisation

**Figure 4:**
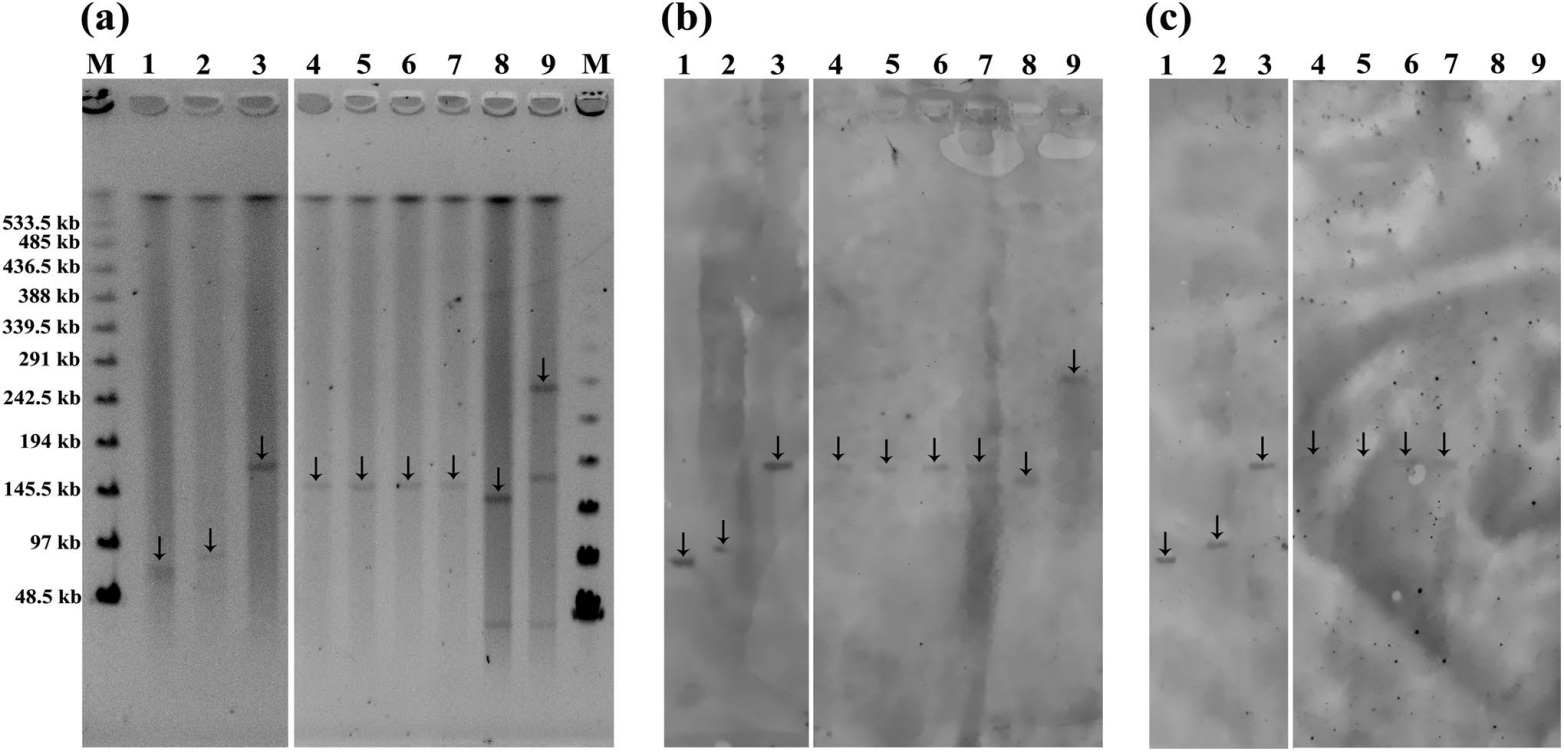
Plasmid profile analysis of nontyphoidal *Salmonella* isolates with conjugative plasmid carrying the *mcr-3* gene together with *bla*_CTX-M-55_ gene by S1-PFGE and Southern blot hybridisation. Representative (**a**) PFGE profiles of total DNA digestion with S1 nuclease, and (**b**,**c**) relative hybridisation with the (**b**) *mcr-3* probe and (**c**) *bla*_CTX-M-55_ probe. Lane 1, plasmid profile analysis of *S.* Choleraesuis strain H-17–072; lane 2, *S.* Choleraesuis strain H-17–073; lane 3, *S.* Choleraesuis strain H-17–053; lane 4, *S.* Typhimurium strain H-07–020; lane 5, *S.* Typhimurium strain H-07–021; lane 6, *S.* Typhimurium strain H-07–037; lane 7, *S.* Typhimurium strain H-07–038; lane 8, *S.* Typhimurium strain H-07–385; lane 9, *S.* Typhimurium strain H-07–388; lane M, CHEF DNA Size Standard-Lambda Ladder (#170–3635), marker labels are in kilo-bases. Arrows indicate the locations of AMR plasmids.

The 22 *mcr-3*-carrying *Salmonella* isolates and two *mcr-1.9*-carrying *Salmonella* isolates were characterised for plasmid profiles by S1-PFGE and the locations of the AMR genes were identified by Southern blot hybridisation using the specific probes for the respective genes. The transferability of *mcr*-carrying plasmid was determined in all *mcr*-carrying *Salmonella* isolates using transconjugation experiment. Of 24 *mcr*-carrying *Salmonella* isolates, 14 isolates successfully transferred *mcr* gene to the recipient, seven of which co-transferred *mcr-3.1* and *bla*_CTX-M-55_ genes. Remarkably, the self-transferable *mcr-1.9*-carrying plasmid, 33 kb-IncX4, was spread among two different clones (E1 and E2) of *S.* 4,5,12:I:- isolated from a stool sample from a patient in Chiang Mai in 2017 (Fig. 3). In addition, the *mcr-3.1* gene was disseminated among at least five genetically unrelated clones (cluster A, C, D, E, and F) via various plasmids of IncA/C, IncA/C-IncFIIs, IncFIIs, and IncFII, with a size ranging from 40–283 kb (Fig. 3). The plasmids displayed high conjugation efficiencies, ranging from 2.3 × 10^–5^ to 1.3 × 10^–2^ colony forming units (CFU) per recipient cell. Of particular note, probe hybridisation revealed that the *mcr-3.1* and *bla*_CTX-M-55_ genes were co-located on the same plasmid in seven of the *mcr-3.1*-carrying *Salmonella* isolates, three of which also co-existed with the *qnrS1* gene (Figs. 3 and 4). The co-dissemination of both *mcr-3.1* and *bla*_CTX-M-55_ genes in *S.* Typhimurium isolates in all cases was mediated by the  ~ 170-kb conjugative plasmid IncA/C, which was spread among two different clones (E5 and E6) of *S.* Typhimurium isolated from a stool and a rectal swab in 2007 (Fig. 4). Moreover, the co-dissemination of these genes in *S.* Choleraesuis isolates was mediated by two multi-replicon plasmids (IncA/C and IncFIIs), which had self-transferable plasmid sizes of 66-kb, 83-kb, and 168-kb (Fig. 4). The dendrogram revealed that these conjugative plasmids were disseminated among at least two genetically unrelated clones (cluster A and F) of *S.* Choleraesuis, isolated from three different bacteremic patients in Chiang Mai during 2017 to 2018 (Fig. 3). Interestingly, the co-transference of colistin and ESC resistance was explored in seven *Salmonella* isolates by conjugation, using the azide-resistant *E. coli* K12 as a recipient. High conjugation efficiencies were demonstrated, ranging from 3.3 × 10^–4^ to 5.0 × 10^–4^ CFU per recipient cell in these resistance plasmids (Table 1), while their transconjugants showed a colistin MIC ranging from 4–8 μg/mL and a ceftriaxone MIC ranging from 64–256 μg/mL. The transconjugants exhibited 16- to 32-fold and 64- to 256-fold increases in the MICs of colistin and ceftriaxone, respectively, compared with those in a recipient (Table 1). Remarkably, most isolates were also co-transferred with the resistance to tetracycline, gentamicin, and chloramphenicol (Table 1). In addition, the recombinant plasmid of MCR-3.1 displayed a slight colistin effect, with a 16-fold increase in the MIC of colistin from 0.25 to 4 μg/mL, compared with those in *E. coli* DH5α containing pBK-CMV alone. The recombinant CTX-M-55 plasmid resulted in a 32-fold increase in the MIC of ceftriaxone from 1 to 32 μg/mL compared with those in *E. coli* DH5α containing pBK-CMV alone (Table 1).

**Table 1:**
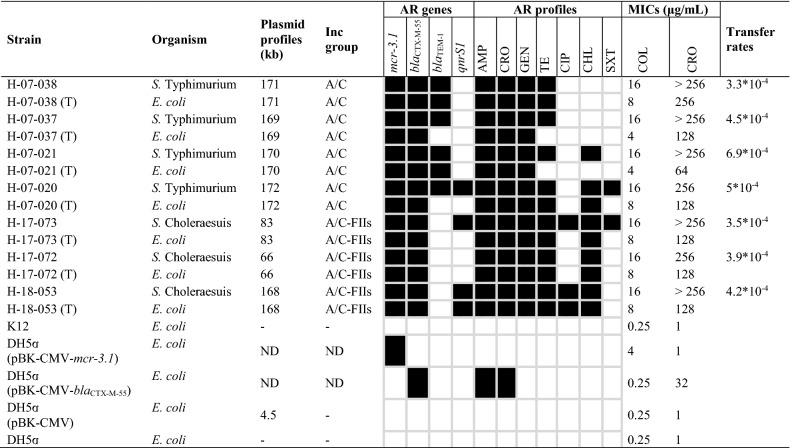
Characterisation of seven *Salmonella* isolates co-carrying *mcr-3.1* and *bla*_CTX-M-55_ genes, and the corresponding transconjugants and transformants.

The alphabet letter (T) represents the corresponding transconjugants. Black squares represent the isolates as being resistant to the indicated antimicrobial agents.

*AMP* ampicillin, *CRO* ceftriaxone, *GEN* gentamicin, *TE* tetracycline, *CIP* ciprofloxacin, *CHL* chloramphenicol, *SXT* trimethoprim-sulfamethoxazole, *COL* colistin, *ND* not determined, *-* not found.

## Discussion

Since the first discovery of the *mcr* gene in China in late 2015^3^, the *mcr-1* gene has rapidly spread globally within Enterobacteriales across the 30 countries in five continents^27^. To date, nine other *mcr* genes have been identified (*mcr-2*^4^, *mcr-3*^5^, *mcr-4*^6^, *mcr-5*^7^, *mcr-6*^8^, *mcr-7*^9^, *mcr-8*^10^, *mcr-9*^11^, and *mcr-10*^12^). In Thailand, a few studies have investigated the *mcr* genes, mostly *mcr-1*, and only in *E. coli* and *Klebsiella pneumoniae* isolates, which found approximately 2% and 1% of isolates during 2014–2018, respectively^28–30^. The *mcr* gene was first recognised in Thailand in three of 179 *E. coli* clinical isolates in Chachoengsao province, Central Thailand, in as early as 2014^30^.

As far as we are aware, this study reports here for the first time a high prevalence of colistin resistance and *mcr* genes in nontyphoidal *Salmonella* isolated from patients in Thailand. The occurrence of *mcr* genes might be more common in *Salmonella* with 11.2% of isolates during 2014–2018 than those previously recognised by other studies, which reported only a low frequency of *mcr*-positive *Salmonella* (about 1%) among clinical isolates during 2012–2015^31^. Remarkably, the current high frequency of *mcr* genes in *Salmonella* isolates was attributed to the dissemination of the *mcr-3* gene. Despite the *mcr-3* gene was first discovered in porcine *E. coli* isolates in China in 2015^5^, it was detected in *Salmonella* isolated from patients in 2007 from our retrospective study of *Salmonella* clinical isolates during 2005–2007 in Thailand. Thus, it should be noted that the *mcr* gene, especially *mcr-3.1*, has already spread in Thailand since at least 2007, the earliest isolation time point of the *mcr*-harbouring isolates among our collection of *Salmonella* clinical isolates. Currently, the *mcr-3* gene has become more widespread and prevalent in *Salmonella*, *S.* Typhimurium, and a monophasic variant of *S.* Typhimurium in particular^21,24,25^. The clonal spread of the MDR ST34 *S.* Typhimurium (including monophasic variants) was observed in China during 2014–2019, during which time the global distribution was confirmed by the high similarity between the genomes of *mcr-3*-harboring *Salmonella* isolated from across four continents^25^. Interestingly, most patients with *mcr-3*-positive *Salmonella* infections from Denmark, Canada, USA, and Australia had a travel history to Thailand, Vietnam, or China, during 2013–2016^25^.

Moreover, we found some AMR plasmids supporting the dissemination of the *mcr-3* gene among diverse genetic backgrounds, including the IncA/C, IncHI2, IncFII, and IncA/C-IncFIIs plasmids. These plasmids have been described in *E. coli* and in few serotypes of *Salmonella* that have mostly been recovered from food animal isolates^25^. Remarkably, two distinct monophasic variants of *S.* Typhimurium isolates carried the *mcr-1.9* gene on IncX4 plasmids with an identical size of 33 kb. In accordance with previous studies, the 33-kb IncX4 plasmid carrying the *mcr-1.9* gene has been identified in unrelated *E. coli* and *K. pneumoniae* isolates from humans and food-producing animals in many countries, including China, Italy, United States, Brazil, and Portugal^32,33^.

The present study reported an increasing frequency of AMR among nontyphoidal *Salmonella* clinical isolates from all six regions of Thailand over the last 8 years, in which more than three-quarters of the isolates showed a MDR phenotype. Remarkably, a significantly higher proportion of *mcr* gene positive isolates were recognised in the ESC-resistant *Salmonella* isolates than in the non-ESC-resistant counterparts. More worrisome, the frequency of colistin and ESC resistance have dramatically increased over recent years and a high proportion of MDR *Salmonella* co-harbouring *mcr-3* and *bla*_CTX-M-55_ genes has been described in this study. Interestingly, the co-localisation of *mcr-3* and *bla*_CTX-M-55_ genes on the same plasmid was recognised in a high proportion of these isolates (7/15, 46.7%), which were attributed to the self-transferable MDR plasmids of the ~170-kb IncA/C (formerly known as IncC) and novel hybrid IncA/C-IncFIIs (size of 66-kb, 83-kb, and 168-kb) plasmids in nonclonal *S.* Typhimurium and *S.* Choleraesuis strains, respectively. The self-transferable IncA/C plasmid, a broad host range plasmid, has previously demonstrated an important role in the dissemination of the *bla*_CTX-M-55_ gene among *S.* Choleraesuis in blood isolates, leading to the high prevalence of ESC resistance in Thailand in recent years^34^. The hybrid IncA/C-IncFIIs plasmid shared the same backbone modules of both IncA/C and IncFIIs (species-specific FII replicons for *Salmonella*) plasmids, where the genetic plasticity of the plasmid probably shaped the dissemination of the resistance determinants, intensifying the spread of MDR and XDR in *Salmonella*. This is similar to a previous study, where an IncC-FII hybrid plasmid played a role in the widespread distribution of ST34 in *S.* Typhimurium and a monophasic variant co-carrying *mcr-3* and *bla*_CTX-M-55_ genes in China or even globally, mediated by IS15DI^25^. The specific genetic background is required for acquisition and maintenance of *mcr*-carrying plasmids.

Our study demonstrates that the *mcr* genes were disseminated through neither plasmid nor clone specific where the clonality analysis revealed a polyclonal spread. However, three indistinguishable *mcr-3.1*-carrying *S*. Typhimurium were identified in two different provinces in 2007, probably a small outbreak at that time. The *mcr-3* has rapidly disseminated and become the most common *mcr* gene among the isolates. The IncA/C plasmid could probably be the major vehicle of the spread of *mcr-3* in Thailand from as early as 2007. Moreover, this study, taken together with previous studies^25^, indicates that the dissemination of the *mcr-*3-carrying MDR *S*. Typhimurium and monophasic serovar could potentially become globally spread and of great concern. Remarkably, to our knowledge the occurrence of *mcr-3* and its co-existence with *bla*_CTX-M-55_ were described for the first time among *S.* Choleraesuis in the present study, all of which were from bacteremic patients. This becomes a clinical concern over the treatment of the invasive salmonellosis. With the highest ability to cause septicemia (OR 44.00; 95% CI 34.28–56.47), *S.* Choleraesuis was previously ranked the most common recovered serotype from bacteremic patients in Thailand^34,35^. Noticeably, a high proportion of colistin-resistant *Salmonella* without the *mcr* gene was observed in this study, underlining chromosomal mutations and other resistance mechanisms. The common chromosomal mutations that have been reported in *Salmonella* involve the PmrA/PmrB and PhoP/PhoQ two-component regulatory systems^36^.

Notably, both colistin and cephalosporins (more specifically ceftiofur) have been extensively used in food animal systems over the past few decades in many countries^37^. Considering that the frequencies of both *mcr-3*-positive *Salmonella* and *Salmonella* co-carrying *mcr-3* and *bla*_CTX-M-55_ genes have arisen over those same time periods, this could be attributed to the co-selection by colistin and/or cephalosporins in both a veterinary field and human clinical setting. This notion was supported by the high agreement between the increasing trends of colistin resistance and ESC resistance over the past 8 years, as demonstrated in the present study. This highlights the urgent need to strengthen an AMR control strategy to prevent further spread, since this poses a threat to global health due to travel and trade in animal products.

## Materials and methods

### Bacterial strains

A total of 724 non-duplicate nontyphoidal *Salmonella* clinical isolates from various hospitals in 32 provinces from different regions of Thailand during 2005–2007 (n = 563) and 2014–2018 (n = 161) were characterised. Clinical isolates used in this study were obtained from a repository collection after standard characterisation and identification as part of the standard care of the patients that was unrelated to the present study. There were 620 isolates obtained from the *Salmonella* and *Shigella* Center, National Institute of Health, Department of Medical Sciences (Nonthaburi, Thailand) and 104 isolates from the Department of Microbiology, King Chulalongkorn Memorial Hospital (Bangkok, Thailand), Queen Savang Vadhana Memorial Hospital (Chonburi, Thailand), Phra Pok Klao Hospital (Chanthaburi, Thailand), and Surin Hospital (Surin, Thailand). Most samples (32.3%) were collected from patients in Bangkok. The common specimen isolated were from blood, stool, rectal swab, and urine, most of which were from blood (58.3% of isolates). The isolates were identified by biochemical characteristics and the serotyping of *S. enterica* was performed according to the Kauffman-White serotyping scheme^38^.

### Antimicrobial susceptibility testing

The antimicrobials ampicillin, ceftriaxone, ceftazidime, gentamicin, tetracycline, ciprofloxacin, nalidixic acid, chloramphenicol, trimethoprim-sulfamethoxazole, ertapenem, and colistin were obtained from Sigma-Aldrich (St. Louis, MO, USA). MICs of the antimicrobial agents were determined by the agar-dilution technique, except the broth-microdilution technique was used for colistin. The Clinical and Laboratory Standards Institute criteria were used for interpreting the MICs^39^.

### Detection of AMR determinants

A total of 724 *Salmonella* isolates were screened for the presence of *mcr-1* to *mcr-9* genes by multiplex PCR using both the primers designed in this study (Supplementary Table S1) and those previously described^9,40^. The DNA sequences of the entire *mcr* genes were determined by PCR amplification and DNA sequencing of the amplicons using the indicated primers (Supplementary Table S1).

All isolates for which MICs of ≥ 2 μg/mL for either ceftriaxone or ceftazidime were further confirmed for the presence of the *bla*_OXA_, *bla*_TEM_, *bla*_SHV_, *bla*_CTX-M_, and *bla*_VEB_ genes by multiplex PCR as previously described^41–43^. Specific CTX-M groups (CTX-M-1, CTX-M-2, CTX-M-9, and CTX-M-8/25 groups) were also investigated by multiplex PCR^44^, as was the presence of plasmid-mediated *amp*C genes, as previously described^45^. The obtained DNA sequences of the entire *bla* genes were determined by PCR amplification and DNA sequencing as previously reported^34^.

A total of 79 ciprofloxacin-resistant *Salmonella* isolates were determined for the quinolone resistance-determining region (QRDR) mutations in the *gyrA* and *parC* genes, and plasmid-mediated quinolone resistance (PMQR) genes. The *gyrA* and *parC* genes were PCR-amplified and sequenced using the previously described primers and method^46^, and the nucleotide sequences were compared to those of *S.* Typhimurium LT2^46^. The presence of the PMQR genes, including *qnrA*, *qnrB*, *qnrC*, *qnrD*, *qnrS*, *aac(6’)-Ib-cr*, and *qepA*, was screened by PCR as previously described^47,48^.

### Clonal analysis

Pulsed-field gel electrophoresis (PFGE) was used to determine the clonal relatedness of the *mcr*-harbouring *Salmonella* isolates using the PulseNet International protocol 2017 from the Centers for Disease Control and Prevention. Total bacterial DNA, in the form of low-melt agarose plugs, was cut with *Xba*I (Fermentas, USA) and separated using a CHEF-Mapper XA PFGE system (Bio-Rad, Hercules, USA). The PFGE patterns were compared by InfoQuest^TM^FP Software, version 4.5 (Bio-Rad, Hercules, USA). The relatedness of the clones was determined using the Dice coefficient, unweighted pair group method with arithmetic means (UPGMA), 1.0% optimisation, and 1.0% band position tolerance, and interpreted based on the percentage similarities and Tenover criteria^49^.

### Transfer of colistin resistance

Twenty-four *mcr*-carrying *Salmonella* isolates were determined for the transferability of colistin resistance by transconjugation experiment using the broth-mating technique and an azide-resistant *E. coli* K12 strain as the recipient, as previously described^50^. Transconjugants were selected from the MacConkey agar plate containing 150 μg/mL of sodium azide and 2 µg/mL of colistin and, confirmed by PCR. The MICs of antimicrobial agents for the donor, recipient, and transconjugant strains were determined by the agar-dilution technique, except the broth-microdilution technique was used for colistin. After that, their MICs were compared. The conjugation efficiencies of these *mcr*-carrying *Salmonella* isolates were then determined.

### Plasmid analysis

Plasmid profiles of all *mcr*-carrying isolates were characterised by PFGE using S1 nuclease (S1-PFGE). The plasmid DNA was first linearised by S1 nuclease, where the total bacterial DNA in low-melt agarose plugs was digested with S1 nuclease (Fermentas, USA) and separated using a CHEF-Mapper XA PFGE system (Bio-Rad, Hercules, USA). A Lambda ladder (Bio-Rad, Hercules, USA) was used as the molecular-weight size marker and InfoQuest^TM^FP Software, version 4.5 was used to estimate the plasmid sizes. The locations of the *mcr* and *bla* genes in the plasmids were determined by Southern blot hybridisation using specific probes. Probe labelling, hybridisation, and detection were performed using the DIG DNA labelling and detection kit (Roche Diagnostics, Indianapolis, IN, USA), according to the manufacturer’s protocols. The major plasmid types found in Enterobacteriales, including FIA, FIB, FIC, HI1, HI2, I1-IƳ, L/M, N, P, W, T, A/C, K, B/O, X, Y, F, FIIA, and X4 replicon types, were detected by PCR-based replicon typing ^51,52^.

### Cloning of AMR genes

The AMR genes, *mcr-3.1* and *bla*_CTX-M-55_ were PCR amplified using the primers shown in Supplementary Table S1. These PCR products were cloned into the pTZ57R/T TA vector (Fermentas, UK) and subcloned into the pBK-CMV expression vector (Stratagene, La Jolla, CA) with EcoRI and ApaI digestion. The recombinant vectors were transformed into *E. coli* DH5α and then selected for transformants on LB agar plates containing 50 μg/mL of kanamycin using BlueWhite colony screening. The selected colonies were confirmed for correct inserts by PCR and DNA sequencing.

### Statistical analysis

The significance of any differences in the AMR profile was determined by Fisher's exact test (two-tailed) using the GraphPad Prism 5 software (GraphPad Software, Inc., La Jolla, CA, USA), accepting significance at the p < 0.05 level.

### Ethical approval

The study protocol was approved by the Mahidol University Central Institutional Review Board (MU-CIRB), Mahidol University (Nakhon Pathom, Thailand) [MU-CIRB 2019/032.0402]. All experiments were performed in accordance with the ethical standards as laid down in the 1964 Declaration of Helsinki and its later amendments and comparable ethical standards.

### Informed consent

For this type of study of anonymised clinical isolates, the requirement for informed consent from patients was waived by the Mahidol University Central Institutional Review Board (MU-CIRB), Mahidol University (Nakhon Pathom, Thailand) [MU-CIRB 2019/032.0402].

## Supplementary Information

Supplementary Information.

## Data Availability

The data specific to the clinical isolates that support the findings of this study are not publicly available because of privacy/ethical restrictions. However, upon reasonable request, the data will be available from the corresponding author T.C.
