# Supplementary material for: Occurrence of mcr-mediated colistin resistance in Salmonella clinical isolates in Thailand
Source: Sci Rep. 2021 Jul 8;11:14170. doi: 10.1038/s41598-021-93529-6 (PMC8266838; doi:10.1038/s41598-021-93529-6)
Supplement: Supplementary file 1 — Supplementary Information. [file 41598_2021_93529_MOESM1_ESM.pdf]

Supplementary information

Table S1

Primers used in this study

Purpose	Primer	Primer sequence (5’- 3’)	Accession number
Screening of *mcr-6*	MCR6_FW	TTATCCGATGGCACAAAAC	MF176240
	MCR6_RV	TGACCTTATCCGCTGTGAC	
Screening of *mcr-8*	MCR8_FW	ACAATCGGCAACATAGCAC	MG736312
	MCR8_RV	ATTACCGGTCAGGCATTCT	
Screening of *mcr-9*	MCR9_FW	TCACTGGCTCACTGGTCAG	MK791138
	MCR9_RV	GCACGGAACGGATATTGCG	
Amplification and	MCR1_SEQ_FW	GCCGCAATTATCCCACCG	KU761326
sequencing of *mcr-1*	MCR1_SEQ_RV	CCCACCGCCCATAATACGAATGG	
Amplification and	MCR3_SEQ_FW	CTGAAATCCTGCTCAGGTCA	MH114596
sequencing of *mcr-3*	MCR3_SEQ_RV	GCCTGAGCCCCCAATAAT	
Amplification and	CTXM55_SEQ_FW	ATGGTTAAAAAATCACTGCGCC	AY458016
sequencing of *bla*$_{CTX-M-55}$	CTXM55_SEQ_RV	TTACAAACCGTCGGTGACGA	
Amplification and	QNRS1_SEQ_FW	TGGAAACCTACAATCATACATATCG	GQ438249
sequencing of *qnrS1*	QNRS1_SEQ_RV	TTAGTCAGGATAAACAACAATACCC	

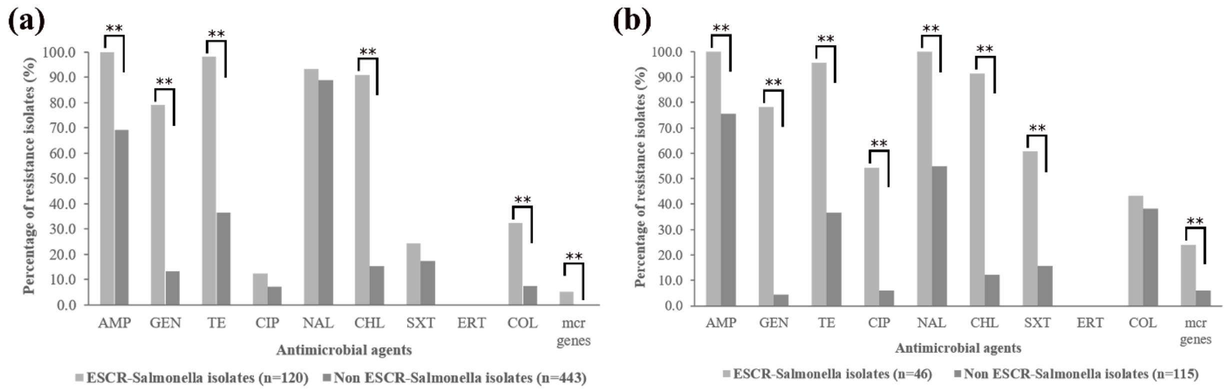

Fig. S1. Comparison of the AMR frequencies between ESC-resistant *Salmonella* and non ESC-resistant counterparts in (a) 2005-2007 and (b) 2014-2018. AMP, ampicillin; GEN, gentamicin; TE, tetracycline; CIP, ciprofloxacin; NAL, nalidixic acid; CHL, chloramphenicol; SXT, trimethoprim-sulfamethoxazole; ERT, ertapenem; COL, colistin; and ESCR, extended-spectrum cephalosporin resistance; *, $p < 0.05$; and **, $p < 0.01$.
